# Supplementary material for: Volatiles from Different Instars of Honeybee Worker Larvae and Their Food
Source: Insects. 2019 Apr 25;10(4):118. doi: 10.3390/insects10040118 (PMC6523346; doi:10.3390/insects10040118)
Supplement: Supplementary file 1 [file insects-10-00118-s001.pdf]

Table S1. Linearity of response for standards. Calibration fitting: y = kx + m [a].

Compound	Linear Range (ng)	Slope (k, Mean ± SD [b], n = 3)	Intercept (m, Mean ± SD [b], n = 3)	Regression (r^2, n = 6)	LOD [c] (ng)	LOQ [d] (ng)	Codes [e] of the Compounds Calculated by This Curve
Aldehyde							
Octanal	0.004–40	0.047 ± 0.0019	0.01±0.0008	0.99982	0.0003	0.0011	a1, a2
Decanal	0.0041–41	0.096 ± 0.0027	0.007±0.0002	0.9974	0.0004	0.0014	a3
Ester							
Methyl tridecanoate	0.0044–44	0.168 ± 0.0047	0.019±0.0041	0.9994	0.00048	0.0016	e1
Hydrocarbon							
Tetradecane	0.0017–17	0.097 ± 0.0061	0.0063±0.00059	0.9997	0.00039	0.0013	h1, h2, h3
Ketone							
6-Methyl-5-hepten-2-one	0.0038–38	0.277±0.0005	0.08±0.0015	0.9992	0.00036	0.0012	k1
Terpenoid							
(*E*)-β-Ocimene	0.005–50	0.282 ± 0.005	0.080±0.008	0.9993	0.00102	0.0034	t1, t2, t3, t4, t5, t6, t7

[a] In the regression equation y = kx + m, y refers to the peak area ratio of target compound to internal standard, x is the concentration ratio of target compound to internal standard, r^2 is the correlation coefficient of the equation; [b] Standard deviation is abbreviated as SD; [c] Limit of detection, S/N = 3; [d] Limit of quantitation, S/N = 10; [e] The codes correspond to the volatile codes listed in Table 1.
